# Effects of enteral nutrition with different energy supplies on metabolic changes and organ damage in burned rats

**DOI:** 10.1093/burnst/tkac042

**Published:** 2022-11-21

**Authors:** Yong-Jun Yang, Sen Su, Yong Zhang, Dan Wu, Chao Wang, Yan Wei, Xi Peng

**Affiliations:** Clinical Medical Research Center, Southwest Hospital, Third Military Medical University (Army Medical University), Chongqing China; Clinical Medical Research Center, Southwest Hospital, Third Military Medical University (Army Medical University), Chongqing China; Institute of Burn Research, State Key Laboratory of Trauma, Burns and Combined Injury, Southwest Hospital, Third Military Medical University (Army Medical University), Chongqing China; Clinical Medical Research Center, Southwest Hospital, Third Military Medical University (Army Medical University), Chongqing China; Institute of Burn Research, State Key Laboratory of Trauma, Burns and Combined Injury, Southwest Hospital, Third Military Medical University (Army Medical University), Chongqing China; Clinical Medical Research Center, Southwest Hospital, Third Military Medical University (Army Medical University), Chongqing China; Clinical Medical Research Center, Southwest Hospital, Third Military Medical University (Army Medical University), Chongqing China; Institute of Burn Research, State Key Laboratory of Trauma, Burns and Combined Injury, Southwest Hospital, Third Military Medical University (Army Medical University), Chongqing China; Shriners Burns Hospital, Massachusetts General Hospital, Harvard Medical School, Boston, MA 02114, USA

**Keywords:** Burn, Energy supply, Enteral nutrition, Hypermetabolic, Organ damage

## Abstract

**Background:**

Enteral nutrition (EN) is an important treatment for burn patients. However, severe gastrointestinal damage caused by major burns often leads to EN intolerance. Trophic EN solves this problem basically, but how to transition from trophic EN to standard EN smoothly is still a challenge in burn clinical nutrition. The aim of this study is to investigate the effects of EN with different energy supplies on metabolic changes, organ damage and prognosis in burned rats.

**Methods:**

Different feeding regimens were designed based on the continuous monitoring of resting energy expenditure in rats. Thirty-two Sprague–Dawley rats were randomly divided into a normal control group, burn +50% REE group, burn +75% REE group and burn +100% REE group. At the end of a nutritional treatment cycle (14th day), nuclear magnetic resonance spectroscopy, blood biochemistry analysis and quantification of subscab bacteria were performed to explore the differences in metabolic changes, degrees of organ damage and prognoses between the groups.

**Results:**

Sixteen metabolites involving seven metabolic pathways were identified from the different energy supply groups. After burn injury, resting energy consumption and body weight loss increased obviously. Meanwhile, weight loss was inversely related to energy supply. The greatest changes in the degree of organ damage, the level of plasma proteins, lipids and endotoxins, as well as the quantification of subscab bacteria were observed in the 50% REE group, followed by the 75 and 100% groups.

**Conclusions:**

Achieving an early balance between energy supply and expenditure is conducive to mitigating metabolic disorders and improving prognosis after burn injury.

Highlights

- Different energy supplies have an obvious effect on metabolic changes and organ damage in burned rats.
- An adequate energy supply is the key factor for alleviating organ damage and reducing the extent of wound infection and mortality.
- Seventy-five percent REE supply in the first week can reduce overfeeding and 100% REE supply in the second week can avoid underfeeding effectively after burns, providing a reasonable energy supply strategy for burned rats.

## Background

Uncontrolled and persistent hypermetabolism is the most notable metabolic feature of severe burn patients. On the one hand, these patients persistently experience a hypercatabolic state, with increased body consumption and an urgent need for nutrition supplementation; on the other hand, they suffer impaired nutrient utilization, i.e. nutrition intolerance [1–3]. Therefore, one of the core clinical issues in this field is how to implement effective nutritional support, especially rational energy supply, for severe burn patients. Insufficient energy intake after burn will not meet metabolic needs, resulting in underfeeding, while excessive energy intake leads to overfeeding and aggravates metabolic complications; both of these conditions can lead to adverse clinical outcomes [2,4,5].

Enteral nutrition (EN), one of the main nutritional support methods for severe burn patients, has attracted considerable attention because of its unique advantages in maintaining the intestinal mucosal barrier. However, burn victims exhibit extensive gastrointestinal congestion, edema and motor dysfunction, which limit the application of EN [6–8]. Trophic EN, which aims to moderately stimulate the gastrointestinal mucosa rather than supply energy, provides a basic solution to this dilemma. A number of clinical trials with critically ill patients have confirmed that trophic EN has nutritional efficacy equivalent to that of conventional EN, significantly reduces gastrointestinal complications and is better tolerated by patients [9–12]. Therefore, trophic EN is widely used in the nutrition therapy of critically ill patients and generally lasts ~1-2 weeks. In this period, the energy supply should be raised to ~80% of the target, below which a poor prognosis will result [13–15]. Zusman *et al*. [16] reported that the relationship between mortality and energy supply in critically ill patients is a U-shaped curve. Mortality is lowest as the caloric intake increases to 70% of target calories, but this is followed by an increase in mortality, particularly as the caloric intake becomes greater than the target. A similar result was obtained in Heyland *et al*.’s study [17], who considered that the optimal early energy supply for critically ill patients should reach 85% of the target energy. Therefore, for critically ill patients, a moderately low caloric strategy in the early stage is beneficial for improving patient prognosis.

The pathophysiological and metabolic characteristics of severe burn patients are not entirely comparable to those of non-burn critically ill patients [18–20]. Clinical evidence of whether trophic EN regimens for critically ill patients can be used in burn patients is lacking. Clinical experience with burn patients has shown that providing adequate nutrition as soon as possible prevents further injury and accelerates rehabilitation [2,9,16]. In addition, studies in critical medicine have also found that achieving a balance between energy supply and expenditure early in treatment helps to reduce energy consumption, maintain organ function and improve the clinical prognosis [21–25]. Therefore, trophic EN should be administered for a limited duration. A smooth transition from trophic EN to conventional EN is a challenge in clinical nutrition. Unfortunately, due to ethical limitations, there is a lack of prospective clinical controlled trials that use different energy supply methods in the continuous treatment of burn patients. Accordingly, this research adopts an animal burn model to explore the effects of different energy-providing regimens on metabolic changes and organ damage and aims to (1) identify a rational feeding method and (2) establish a theoretical basis for future clinical nutrition research.

## Methods

### Experimental animals

Healthy specific pathogen-free-grade male Sprague–Dawley rats, weighing 250 ± 10 g, were purchased from the Animal Experimental Center of Daping Hospital, Third Military Medical University, China. The rats were provided with food and water *ad libitum* for 1 week before the experiment. The feeding room was controlled at a temperature of 25°C and 40% relative humidity under a 12-h light/dark cycle. All animal experiments were performed according to the National Animal Welfare Guidelines and approved by the Institutional Animal Ethics Committee of the Third Military Medical University, Approved Agreement Number AMUWEC2020014.

### Preparation of the animal burn model and nutritional treatment regimens

#### Burn model and grouping

Thirty-two rats were divided into four groups of eight according to the random number table method: the normal control group (sham burn group) (C group), burn +100% resting energy expenditure (REE) supplementation group (100% REE-Sup group), burn +75% REE-Sup group and burn +50% REE-Sup group. The rats were fasted for 12 h before burn injury. All rats were given pentobarbital (40 mg/kg) for anesthesia and buprenorphine (1 mg/kg body weight) for analgesia. After the hair on the back was shaved and the rats were weighed, the shaved area was immersed in 95°C hot water for 15 s to cause grade III burns on 20% of the body surface area. Fluid resuscitation was performed after injury by intraperitoneal injection of lactated Ringer’s solution at 40 ml/kg. Each animal was housed in a single cage and the wound was coated twice a day with iodophor to prevent infection. The rats in the C group were anesthetized and immersed in 37°C warm water for 15 s but were not resuscitated.

#### Nutritional treatment regimens

**Figure 1.**
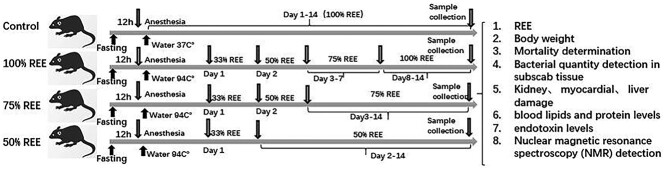
A schematic the main periods of experiment. Different energy supplies can significantlyaffect the metabolic pattern and reduce organ damage in burned rats. *REE* resting energy expenditure

For daily energy supply and nutritious food preparation, the calories supplied to each animal were based on the daily measured REE value and conversion ratios. Based on the polypeptide enteral nutrition preparation Peptisorb (Nutricia Pharmaceutical Co., Ltd, The Netherlands), the energy provision ratio of carbohydrates, proteins and fats was adjusted to 60%, 20%, 20% with glucose powder, amino acid powder (Chongqing Yaoyou Pharmaceutical Co., Ltd, China) and medium long-chain fatty acid emulsion (Fresenius Kabi Huarui Pharmaceutical Co., Ltd, China), respectively, as detailed in Table S1 (see online supplementary material). The prepared rat food was dried at low temperature, cut into pieces and weighed for later use. Nutrition was provided by gavage for 1–2 days postburn, and then the rats were allowed to eat freely. The burned rats were divided into three groups according to the amount of energy supplied as follows. For the 100% REE-Sup group, 33% of the REE was supplied on day 1, 50% on day 2, 75% on days 3–7 and 100% on days 8–14 after injury. For the 75% REE-Sup group, 33% of the REE was supplied on day 1, 50% on day 2 and 75% on days 3–14 after injury. For the 50% REE-Sup group**,** 33% of the REE was given on the first day after injury and 50% on days 2–14 days after injury. For the C group, 100% of the REE was given each day. Daily REE measurements, actual energy supply data, and intake of food and amino acids are showed in Tables S2–S5 (see online supplementary material). The design scheme of the whole experiment are in Figure 1.

### Test indicators

#### REE measurement

REE was measured using indirect calorimetry by measuring respiratory gas exchange with equipment from Columbus Instruments (OH, USA). Rats were placed in a Plexiglas metabolic chamber (4 l volume). The air inlet and outlet contained a calcium sulfate column to desiccate the inhaled and exhaled air. The airflow rate was monitored continuously for 10 min, which covered six cycles (lasting for 60 min), and O_2_ consumption and CO_2_ production were calculated by multiplying the airflow rate by the differences in the inhaled and exhaled O_2_ and CO_2_ concentrations, respectively. Based on these values, the difference in O_2_ intake (DO_2_) was used to calculate CO_2_ (DCO_2_) output and REE using Oxymax software (Columbia Instruments, OH, USA). The REE was calculated before injury and on postburn days 1, 3, 7 and 10 using the following equation: REE (kcal) = [3.94 × DO_2_ (l/min) + 1.1 × DCO_2_ (l/min)] × 1440 × 4.184.

#### Body weight measurement

Individual animals were weighed every morning using an electronic balance with 1/1000 precision (Sartorius, Germany) and the values were recorded.

#### Bacterial quantity detection in subscab tissue

On day 14 after injury, the rats were sacrificed, the back scab was cut and ~0.3 g of the subscab tissue on the back of each animal was cut with a sterile scalpel. After weighing, the tissue was placed in a bacterial culture gel tube for quantitative analysis of bacteria.

### Mortality determination

During the 14-day period of animal observation, the time point of death was recorded and a death–time curve was obtained. Mortality rate (%) = the number of dead animals/the number of participating experimental animals in each group × 100%.

### Serum biochemical indicators

On day 14 after injury, 5–6 ml of blood was drawn from the abdominal aorta under anesthesia and analgesia, 1 ml of the blood was anticoagulated with sodium citrate for nuclear magnetic resonance (NMR) spectroscopy detection and the remaining 4–5 ml was transferred to Ethylene Diamine Tetraacetic Acid (EDTA) tubes for anticoagulation. The tubes were held at room temperature for 30 min and then centrifuged at 4°C at 3000 rpm for 10 min. The serum was collected and stored at −80°C for centralized detection of liver and kidney function, myocardial enzyme spectrum, blood lipids, protein content and endotoxin levels. Testing indicators included the following. Kidney damage: blood urea nitrogen (BUN), creatinine (CrEAT) and uric acid (UA); myocardial damage: lactate dehydrogenase (LDH), α-hydroxybutyrate dehydrogenase (α-HBDH) and creatine kinase isoenzyme MB (CK-MB); liver damage: alanine aminotransferase (AST), aspartate aminotransferase (ALT), glutamyltransferase (GGT), alkaline phosphatase (ALP), total bilirubin (TBIL) and total bile acid (TBA); blood lipids and protein levels: total cholesterol (Tch), triglycerides (TGs), high-density lipoprotein cholesterol (HDL-C), low-density lipoprotein cholesterol (LDL-C), albumin (Alb) and total protein (TP); and endotoxin levels: lipopolysaccharide (LPS), which was quantified by the limulus amebocyte lysate (LAL) test according to the manufacturer’s instructions. Briefly, 100 μl of each sample was added to 100 μl of the limulus reagent (Zhanjiang A&C Biological, China) and the mixture was dissolved in LPS-free water and the reaction was allowed to proceed at 37°C for 60 min in an ATI 320-06 Kinetic Tube Reader (Lab Kinetics Ltd, China). The dynamic turbidity of the limulus reagent was measured to quantify LPS levels.

### Metabolic testing

To observe the effect of different energy supplies on changes in the metabolic pattern of the body after burn, metabolites in rat plasma were detected by NMR spectroscopy and differences between different groups were determined by multivariate statistical analysis. After 14 days of burn, 1 ml of anticoagulant blood was taken from each of the above four groups of rats and centrifuged at 4°C and 16 000 rpm for 10 min, and then 450 μl of plasma was placed into an NMR tube, followed by the addition of 50 μl of heavy water (D_2_O) and thorough shaking for 120 s. Next, the specimen was allowed to stand for 10 min and then placed in a 600-MHz NMR spectroscopy device (Bruker Biospin, DRX, Germany) for measurement and analysis.

### Primary and secondary indicators

Among the above indicators, there are four primary observations, specifically REE, weight change, subscab bacterial quantification and mortality, with the others being secondary indicators.

### NMR spectrum data analysis

Plasma ^1^H-NMR data were imported into MestReNova 12.0.1 software (Mestrelab Research, Santiago de Compostela, Galicia, Spain) for analysis. Before assigning chemical shifts, all spectra were subjected to baseline and phase correction. Chemical shifts of the plasma metabolite spectrum were determined by referring to the methyl resonance peak of lactic acid at δ 1.32. The chemical shift region between 0 and 8 ppm was subdivided into 2000 intervals with a width of 0.004 ppm. The absorption spectrum of water with a chemical shift between 4.7 and 5.1 ppm was removed [26]. Finally, the data were normalized to eliminate differences related to dilution, volume or mass between samples. The same total integral value was assigned to each spectrum before analysis.

The data obtained were imported into SIMCA-P 14.1 (Umetrics, Umea, Sweden) software for principal component analysis (PCA) and orthogonal partial least-squares discriminant analysis (OPLS-DA) [27]. The OPLS-DA model was subjected to random permutations 200 times, and its *R*^2^ and *Q*^2^ were calculated for evaluation. The characteristics of metabolites with intergroup differences were determined based on the *S*-plot curve of the OPLS-DA model and the score for variable importance in projection (VIP > 1) [28]. The characteristics of the screened metabolites were identified in the original spectra using Chenomx NMR 8.5 (Chenomx, Edmonton, Canada) [29,30]. The metabolites were analyzed using the pathway topology search tool in MetaboAnalyst 5.0 (MetPA).

### Statistical analysis

Shapiro–Wilk tests were performed to determine if continuous variables were normally distributed. Normally distributed continuous variables are reported as mean ± standard deviation and non-normally distributed continuous variables are reported as median with interquartile ranges. The two groups were compared using an independent samples t-test and Bonferroni correction (continuous variables with normal distribution and homogeneity of variance) or Mann–Whitney *U* test (continuous variables with a non-normal distribution or heterogeneity of variance). One-way analysis of variance (ANOVA) with Bonferroni correction was performed for comparisons between multiple groups. The changes in the repeated measures were statistically analyzed using two-way repeated measures ANOVA and Bonferroni’s test for multiple comparisons. Categorical variables were expressed as frequencies and percentages compared using Fisher’s exact test. Kaplan–Meier curves were used to analyze the survival time for each group and were further analyzed with the log-rank test. In all analyses, *p* < 0.05 was considered to indicate statistical significance. All analyses were performed using SPSS 25.0, GraphPad 8.0 and R, version 4.0.

## Results

### Effects of different energy supplies on metabolic patterns

**Figure 2.**
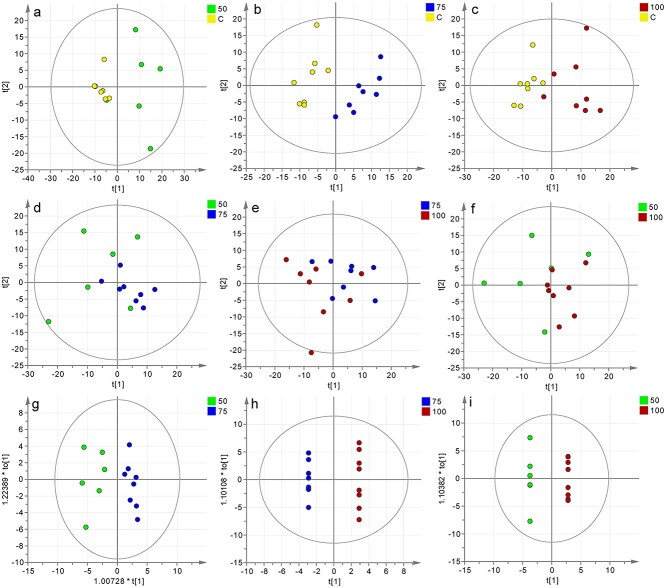
Influence of different energy supplies on metabolic patterns. (**a**) PCA score chart of 50% REE-Sup and C group (*R*^2^ X = 0.647, *Q*^2^ = 0.229). (**b**) PCA score chart of the 75% REE-Sup and the C group (*R*^2^ X = 0.612, *Q*^2^ = 0.115). (**c**) PCA score chart of 100% REE-Sup group and C group (*R*^2^ X = 0.644, *Q*^2^ = 0.101). (**d**) PCA score chart of 50% and 75% REE-Sup group (*R*^2^ X = 0.597, *Q*^2^ = 0.138). (**e**) PCA score chart of 75% and 100% REE-Sup group (*R*^2^ X = 0.525, *Q*^2^ = 0.097). (**f**) PCA score chart of 50% and 100% REE-Sup group (*R*^2^ X = 0.583, *Q*^2^ = 0.082). The score t[1] in the PCAmodel was first principal componentsthat explains the largest variation of the variables space, and the score t[2] was second principal component (a–f). (**g**) OPLS-DA score chart of 50% and 75% REE-Sup group (*R*^2^X = 0.561, *R*^2^Y = 0.911, *Q*^2^ = 0.656). (**h**) OPLS-DA score chart of 75% and 100% REE-Sup group (*R*^2^ X = 0.936, *R*^2^ Y = 1, *Q*^2^ = 0.945). (**i**) OPLS-DA score chart of 50% and 100% REE-Sup group (*R*^2^ X = 0.955, *R*^2^ Y = 1, *Q*^2^ = 0.993). The score t[1]in the OPLS-DA model was the principal components of the OSC process todiscriminate between the two classes. The score to[1] was the orthogonalcomponent of the OSC process, it expressed within class variability (g–i). *REE* resting energy expenditure, *REE-Supp *resting energy expenditure supplementation group, *PCA* principal component analysis, *OPLS-DA* orthogonal partial least-squares discriminant analysis, *C* control group

The PCA score plot showed a significant difference in metabolic patterns between the normal animals and the burned animals (Figure 2a–c), but the three groups of burned rats with different energy supplies could not be completely distinguished (Figure 2d–f). By maximizing covariance between the measured data and the predictive classifications, OPLS-DA analysis between the 50%, 75% and 100% REE-Sup groups was performed to investigate the metabolic patterns of different energy supplies. Comparison of 50 *vs* 75%, 75 *vs* 100% and 50 *vs* 100% all showed clear separation (Figure 2g–i).

**Figure 3.**
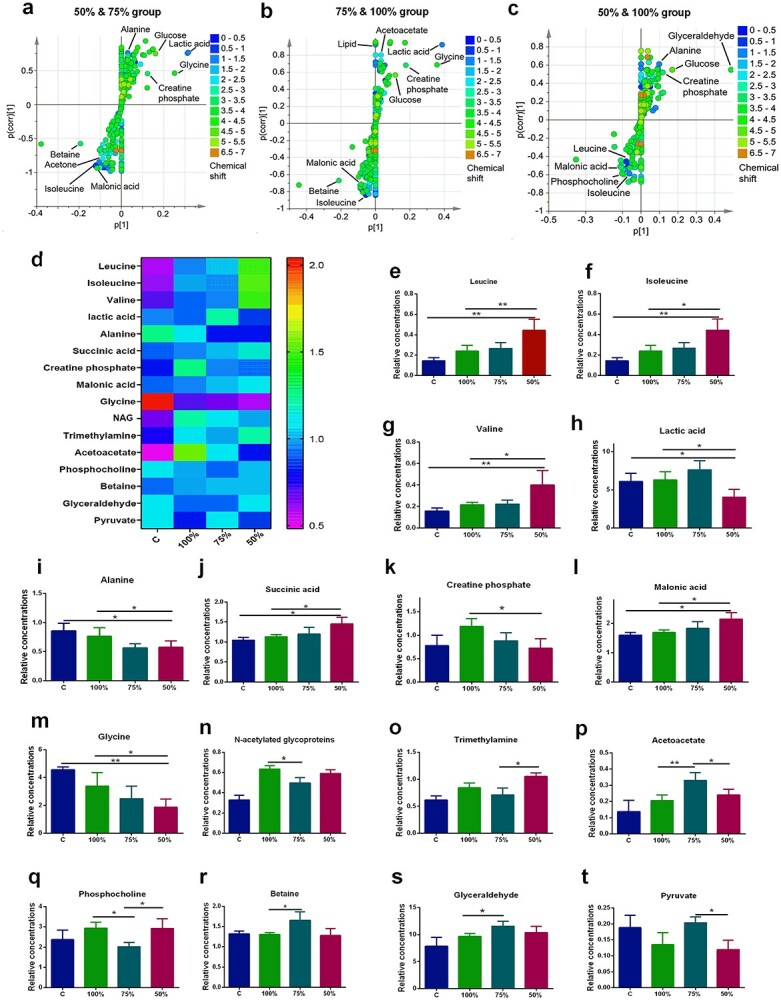
Comparative analysis of metabolic patterns between 100%, 75% and 50% REE-Sup groups. The *S*-plot of OPLS-DA model and some differential metabolites: (**a**) 50% and 75% REE-Sup groups, (**b**) 75% and 100% REE-Sup groups, (**c**) 50% and 100% REE-Sup groups. (**d**) Heatmap of 16 different metabolites in each group (normalized). The 100%, 75% and 50% group nutritional support difference metabolite analysis: (**e**) leucine, (**f**) isoleucine, (**g**) valine, (**h**) lactic acid, (**i**) alanine, (**j**) succinic acid, (**k**) creatine phosphate, (**l**) malonic acid, (**m**) glycine, (**n**) NGA, (**o**) trimethylamine, (**p**) acetoacetate, (**q**) phosphocholine, (**r**) betaine, (**s**) glyceraldehyde and (**t**) pyruvate. *n* ≥ 6 per group, ^*^p < 0.05, ^*^^*^p < 0.01. *REE-Supp* resting energy expenditure supplementation, *OPLS-DA* orthogonal partial least-squares discriminant analysis, *NAG* N-acetylated glycoproteins, *C* control group

**Figure 4.**
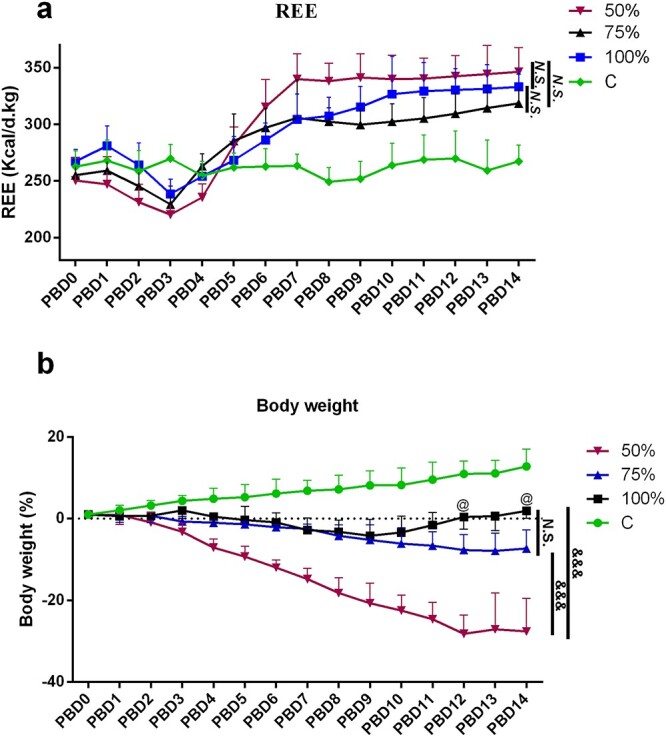
Effect of different energy supplies on energy expenditure and body weight 14 days after burn. (**a**) REE, (kcal/d/kg) of 50%, 75% and 100% REE-Sup groups compared with control group. Two-way repeated measures ANOVA revealed that energy supply (*p* < 0.001) and time (*p* < 0.001) both had a statistically significant effect on energy consumption. There was statistically significant interaction between energy supply and time (*p* < 0.001). Mauchly’s test of sphericity, *p* < 0.05. Bonferroni’s test for multiple comparisons: C *vs* 50% REE-Sup groups, *p* = 0.007; 50% *vs* 75% REE-Sup groups, *p* = 0.987; 75% *vs* 100% REE-Sup groups, *p* = 1.000; 50% *vs* 100% REE-Sup groups, *p* = 1.000. (**b**) Body weight loss of 50%, 75% and 100% REE-Sup groups compared with control group. Energy supply (*p* < 0.001), time (*p* < 0.001), interaction effect between energy supply and time (*p* < 0.001) both had a statistically significant effect on weight loss. Mauchly’s test of sphericity, *p* < 0.05. Bonferroni’s test for multiple comparisons: C *vs* 50% REE-Sup groups, *p* < 0.001; 50% *vs* 75% REE-Sup groups, *p* < 0.001; 75% *vs* 100% REE-Sup groups, *p* = 0.505; 50% *vs* 100% REE-Sup groups. *n* ≥ 6 per group. Body weight at the same time point for different energy supply groups: 75% *vs* 100% REE-Sup group @*p* < 0.05, &&&*p* < 0.001. *N.S.* no significance, *PBD* post burn day, *REE* resting energy expenditure, *ANOVA* analysis of variance, *C* control group

The differential metabolites were identified by pairwise comparisons between 50%, 75% and 100% Sup-REE group groups. Standard seven-fold cross-validation and permutation (200 cycles) were performed to evaluate the stability of the OPLS-DA models (Figure S1a–c, see online supplementary material). The *S*-plot curve was used to identify significantly altered metabolites, which should be farther away from the origin and located in the upper right or lower left quadrant (Figure 3a–c). The significant differential metabolites were identified from different groups by using the *S*-plot curve with significant VIP values >1 and *p*-values <0.05 (Table S6, see online supplementary material). The metabolites were leucine, isoleucine, valine, lactic acid, alanine, succinic acid, creatine phosphate, malonic acid, glycine, N-acetylated glycoproteins (NAG), trimethylamine, acetoacetate, phosphocholine, betaine, glyceraldehyde and pyruvate. The results showed that the levels of leucine, isoleucine, valine, succinic acid, malonic acid, NAG, trimethylamine, acetoacetate and glyceraldehyde in the three burn groups were all higher than those in the C group. Alanine and glycine levels were obviously lower than those in the C group, but they increased with the energy supply. Lactic acid and malonic acid in the 100% REE-Sup group, and pyruvate and creatine phosphate in the 75% REE-Sup group were closest to that in the C group (Figure 3d–t). These differential metabolites in the C group and in the three energy supply groups suggest the amelioration by 75% and 100% energy supply of the disturbed metabolism of burn mice.

MetaboAnalyst 5.0 was used to investigate the metabolic pathways involved in the functional mechanisms of different energy supplies. Seven potential functional pathways, summarized in Table S7 and Figure S2 (see online supplementary material), including glycine, serine and threonine metabolism, butanoate metabolism, citrate cycle (tricarboxylic acid cycle), pyruvate metabolism, glycolysis/gluconeogenesis, glyoxylate and dicarboxylate metabolism, synthesis and degradation of ketone bodies, all exhibited significant changes.

### Effects of the different energy supplies on REE and body weight loss

The results showed that REE increased obviously in the three burned groups and all were higher than that of group C (Figure 4a). Two-way repeated measures ANOVA revealed that energy supply and time, and the interaction between energy supply and time both had a statistically significant effect on energy consumption. Energy consumption increased significantly from day 3–7 in the 50%, 75% and 100% REE-Sup groups. Further, Bonferroni’s test for multiple comparisons found that the 50% group had the greatest energy consumption relative to the C group. However, there was no significant energy consumption difference between mice that received 50%, 75% and 100% REE-Sup group energy supply (Figure 4a). After burn injury, the body weights of rats showed a decreasing trend, and different energy supplies had a significant impact on body weight. Weight loss increased significantly with burn progress in both the 50 and 75% REE-Sup groups. Bonferroni’s test for multiple comparisons found that the 50% REE-Sup group had the greatest weight loss relative to the other groups. However, there was no significant weight loss difference between the 75% and 100% REE-Sup groups (Figure 4b). These findings showed that giving energy with 75% and 100% REE supplementation could relieve weight loss after burn, while there was no significant energy consumption difference between the 50%, 75% and 100% groups.

### Effects of energy supply on blood lipid and protein levels in plasma

**Figure 5.**
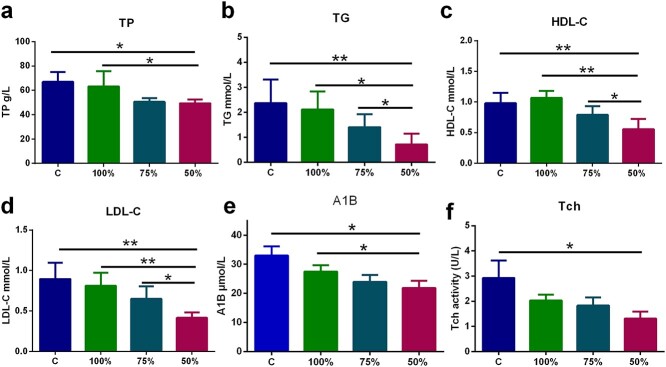
The effect of different energy supply on the blood lipid and protein level in plasma 14 days after burn. Comparing the 50% group with the 100% group, there are significant differences in (**a**) TP, (**b**) TG, (**c**) HDL-C, (**d**) LDL-C, and (**e**) Alb. Compared with the 50% group, (b) TG, (c) HDL-C, (d) LDL-C were remarkably higher in the 75% one than in the 50% group,while (a) TP, (e) Alb, and (**f**) Tch were not Significant differences. *n* ≥ 6 pergroup, ^*^p < 0.05, ^*^^*^p < 0.01. *Alb* albumin, *TP* total protein, *Tch* totalcholesterol, *TGs* triglycerides, *HDL-C* high-density lipoprotein cholesterol, *LDL-C* low-density lipoprotein cholesterol, *C* control group

Fourteen days after burn injury, plasma levels of Tch, TGs, HDL-C, LDL-C, Alb and TP were remarkably lower in the burned rats than in the normal controls, suggesting that severe burn injury can cause abnormalities in lipid and protein metabolism (Figure 5a–f). In addition, different energy supplies affected blood lipid and protein metabolism in the burned rats. The levels of Tch, TG, HDL-C, LDL-C, Alb and TP in the 100% group were remarkably higher than those in the 50% REE-Sup group, and the changes in the 75% REE-Sup group ranged between those in the 100% and 50% groups (Figure 5a–f). These results suggest that 100% REE supplementation had a better effect on stabilizing blood lipids and protein metabolism than 75% or 50%.

### Effects of energy supply on the degree of organ damage in burned rats

**Figure 6.**
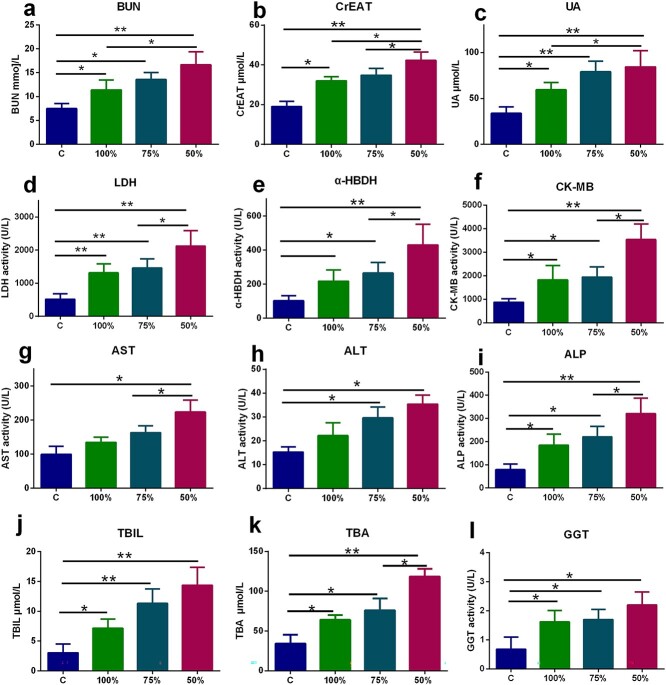
Effect of energy supply on the degree of organ damage 14 days after burn. Comparing the 100 with the 50% REE-Sup group, the 100% group significantly inhibits the increase of (**a**) BUN, (**b**) CrEAT, (**c**) UA, (**d**) LDH, (**e**) *α*-HBDH, (**f**) CK-MB, (**g**) AST, (**h**) ALT, (**i**) ALP, (**j**) TBIL, and (**k**) TBA. Comparing the 75% REE-Supgroup with the 50% REE-Sup group, the 75% one can significantly inhibit theincrease of (b) CrEAT, (d) LDH, (f) CK-MB, (e) α-HBDH, (g) AST, (i)ALP, and (k) TBA. No significant difference in (**l**) GGT between 100%, 75%, and 50% REE groupsfor comparison.*n*  ≥ 6 per group, ^*^p < 0.05, ^*^^*^p < 0.01. *BUN* blood urea nitrogen, *CrEAT* creatinine, *UA* uric acid, *LDH* lactate dehydrogenase, *α-HBDH* α-hydroxybutyrate dehydrogenase, *CK-MB* creatine kinase isoenzyme MB, *ALT* alanine aminotransferase, *ALP* alkaline phosphatase, *AST* aspartate aminotransferase, *GGT* glutamyltransferase, *TBIL* total bilirubin, *TBA* total bile acid,* REE-Supp* resting energy expenditure supplementation group, *C* control group

The experimental results showed that the organs of the rats were remarkably damaged after burn, and the indexes reflecting damage of the kidney, myocardium and liver were significantly higher than those in the normal group. Different energy supplies had different effects on organ damage in burned rats. The 100% energy supply significantly reduced BUN, CrEAT, and UA (Figure 6a–c) levels and LDH, α-HBDH, CK and CK-MB (Figure 6d–f) levels, respectively reflecting kidney damage and myocardial damage, and reduce AST, ALT, GGT, ALP, TBIL and TBA (Figure 6g–l) levels that reflect liver damage, with obviously better results than the 75% and 50% REE-Sup groups. Comparison of the three groups showed that the 100% REE-Sup group had the lowest degree of organ impairment, followed by the 75% and 50% groups. These results suggest that underfeeding is allowed from 1-7 days after burn injury and that providingfull amount of energy supplied according to actual energy consumption at 7 days after burn injury can effectively reduce the degree of organ damage.

### Effects of energy supply on mortality rate and infection

**Figure 7.**
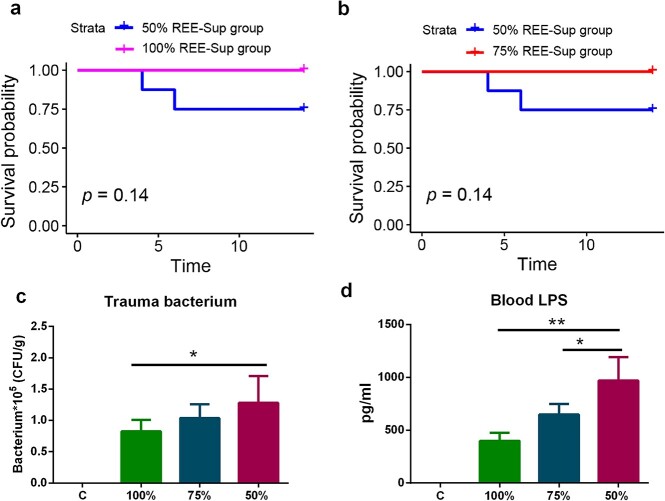
Effect of different energy supply on the mortality and infection of rats 14 days after burn. Kaplan–Meier survival curves for each group, (**a**) 50% *vs* 75% REE-Sup, *p* = 0.14, (**b**) 50% *vs* 100% REE-Sup, *p* = 0.14. Compared with the 50% REE-Sup group, the 100% group can remarkably inhibit the growth of bacteria (**c**) on the wound surface of rats and reduce the blood endotoxin content (**d**). For the 75% compared with the 50% REE-Sup group, the 75% group can significantly reduce the blood toxin (d) content. *n* ≥ 6 per group, ^*^p < 0.05, ^*^^*^p < 0.01. *LPS* lipopolysaccharide,* CFU *colony-forming unit, *REE-Supp* resting energy expenditure supplementation group, *C* control group

The experimental results showed that energy supply had an obvious impact on the mortality rate of rats. No deaths were noted in the 75% and 100% REE-Sup groups, while the mortality rate in the 50% group was 25%. However, there was no significant survival difference between the 50%, 75% and 100% REE-Sup groups analyzed with the log-rank test (Figure 7a, b). Correspondingly, the quantification of subscab bacteria from the 100% REE-Sup group was remarkably lower than that in the 75% and 50% groups (Figure 7c). Plasma endotoxin content was highest in the 50% REE-Sup group, followed by the 75% and 100% groups (Figure 7d). These results suggest that adequate energy supply can effectively reduce the degree of wound infection and mortality in burned animals.

## Discussion

Nutrition therapy is an important component of medical treatment in burn care [1,31,32]. However, appropriate nutrition support is a major challenge in burn patients, due to increased nutritional requirements as well as nutritional intolerance [3,33–35]. Therefore, this study used an animal burn model to observe the effects of different energy supply methods on metabolism, organ damage and prognosis. The results indicated significant nutrition intolerance, such as diarrhea, vomiting and reflux, in the first week of 100% REE supplementation after burn, and ~75% REE supplementation was more appropriate at this stage. From the second week onward, energy should be supplied according to actual consumption. A prolonged period of substandard energy supply aggravates metabolic disorders and organ damage after burn injury, leading to poor outcomes. Therefore, with due consideration of the body’s tolerance, the nutrition supply should be increased at a rapid rate to achieve an early balance between energy consumption and supply.

Metabolomics studies have found that different energy supply methods significantly influence metabolic patterns after burn injury. Sixteen metabolites involved in seven metabolic pathways were identified from different energy supply groups, including the metabolic pathways of proteolysis, gluconeogenesis, glycolysis and ketone bodies; the most significant differences were observed in the proteolysis pathway. In the three energy supply groups, the 50% REE-Sup group had remarkably higher plasma levels of branched-chain amino acids (leucine, isoleucine and valine) than the control group and the other two burn groups. The amino acids released from skeletal muscle proteolysis after burn are mainly used for synthesizing acute-phase protein and glucose (gluconeogenesis), which is the body’s protective response to trauma [36]. However, if catabolism is excessive and prolonged, it depletes the body’s energy reserves, resulting in a poor outcome [37,38]. Our research confirms that prolonged deficiencies in energy supply will lead to continuous skeletal muscle depletion, while higher energy intake is beneficial in alleviating hypercatabolism after burn injury. Combining the changes in REE and body weight, the rats in the 50% REE-Sup group exhibited significantly higher energy expenditure than the other two groups, and their body weight loss was the most pronounced; these changes were accompanied by a marked reduction in plasma protein, triglyceride and cholesterol levels. These results provide ample evidence that prolonged deficiencies in energy supply aggravate hypercatabolism after burn trauma and lead to progressive muscle atrophy.

This study further found that low energy intake exacerbated organ damage post-burn, with the most extensive myocardial, liver and kidney damage observed in the 50% REE-Sup group and the least extensive damage observed in the 100% REE-Sup group among the three energy supply groups. Burn stress, ischemia/hypoxia and uncontrolled inflammation are major contributors to organ damage; additionally, malnutrition can exacerbate organ damage and inhibit tissue repair [9,39]. Clinical studies have found that energy supply adequacy was closely related to organ damage, immune function and wound healing [40,41]. Chronic undernutrition can lead to inadequate cellular energy synthesis, aggravate cellular damage, suppress immune function and impede tissue repair, leading to poor prognosis [41–43]. In the present study, the rats in the 50% REE-Sup group not only exhibited more severe organ damage, but also had significantly higher wound infection, endotoxin levels and mortality than the rats in the other two burn groups. Therefore, early achievement of energy balance helps to reduce organ damage, wound infection risk and even mortality.

The nutritional program of burn patients should be adjusted over time, based on the burn course and the patient’s status [31,44]. The relatively low energy supply in the early stages of burn treatment is due to the patient’s intolerance of exogenous nutrients and the presence of large amounts of endogenous nutrients induced by hypercatabolism [9,13,17]. At this time, energy supply according to energy expenditure will lead to overnutrition. Once the patient’s internal environment has stabilized and hypercatabolism is reduced, the exogenous energy supply should be increased in a timely manner, otherwise undernutrition will likely result [9,45]. Based on the results of this study and in light of our clinical experience, we believe that most severe burn patients (excluding geriatric patients or those with underlying disease) are less nutrition intolerant than critically ill patients with non-burn injuries. Therefore, the permissible low-calorie strategies should not be used for long periods of time in severe burn patients. In the early stages of burn treatment, aggressive therapeutic measures should be taken to improve the internal environment and maintain gastrointestinal function to allow early transition from trophic EN to conventional EN, and the achievement of target energy supply levels should occur within 2–3 weeks post-burn if possible.

Although this study determined the optimal protocol of energy supply for burned rats, another important issue of clinical nutrition, the nutritional formula, was not explored. Thus, to determine the optimal energy supply scheme, we will next explore the optimal nutritional formula. In addition, whether a nutritional regimen optimized for animal models is suitable for human burn patients still requires verification through prospective randomized controlled trials.

## Conclusions

Different energy supply methods can markedly affect metabolic patterns and burn prognosis. A higher energy supply is conducive to mitigating metabolic disorders after burn injury, reducing weight loss and organ damage and improving burn patient prognosis. In the three dose groups in this study, the optimal EN regimen was to supply rats with energy at 75% of REE in the first week post-burn and 100% of REE in the second week.

## Abbreviations

Alb: Albumin; ALP: Alkaline phosphatase; ALT: Alanine aminotransferase; AST: Aspartate aminotransferase; BUN: Blood urea nitrogen; CrEAT: Creatinine; CK-MB: Creatine kinase isoenzyme MB; GGT: Glutamyltransferase; α-HBDH: α-Hydroxybutyrate dehydrogenase; HDL-C: High-density lipoprotein cholesterol; LAL: Limulus amebocyte lysate; LDH, Lactate dehydrogenase; LDL-C: Low-density lipoprotein cholesterol; LPS: Lipopolysaccharide; NAG: N-Acetylated glycoproteins; NMR: Nuclear magnetic resonance; OPLS-DA: Orthogonal partial least-squares discriminant analysis; PCA: Principal component analysis; REE: Resting energy expenditure; TBA: Total bile acid; TBIL: Total bilirubin; TP: Total protein; Tch: Total cholesterol; TGs: Triglycerides, UA: Uric acid; VIP: variable importance in projection; EDTA: Ethylene Diamine Tetraacetic Acid; ANOVA: Analysis of Variance；CFU: colony-forming unit.

## Ethics approval and consent

All animal experiments were performed according to the National Animal Welfare Guidelines and approved by the Institutional Animal Ethics Committee of the Third Military Medical University, Approved Agreement Number AMUWEC2020014. 

## Funding

This work was supported by the National Natural Science Foundation of China (No. 81971838), Clinical Research Foundation of TMMU (No. 2018XLC2006) and Innovative Leading Talents Project of Chongqing (NO. CQYC20210303 286).

## Authors’ contributions

XP designed the trial and control the process. YJY, SS and YZ participated in relevant animal experiments and obtained specimens and data. YZ, SS, DW, YW and YJY obtained test data. YJY and SS participated in statistical analysis and drafted the manuscript. All authors discussed the results and commented on the manuscript.

## Conflicts of interest

The authors declare no conflict of interest.

## Supplementary Material

Supporting_Information_tkac042
