# Supplementary material for: Effects of enteral nutrition with different energy supplies on metabolic changes and organ damage in burned rats
Source: Burns Trauma. 2022 Nov 21;10:tkac042. doi: 10.1093/burnst/tkac042 (PMC9678637; doi:10.1093/burnst/tkac042)
Supplement: Supporting_Information_tkac042 [file supporting_information_tkac042.docx]

Supporting Information

**Effects of enteral nutrition with different energy supplies on metabolic changes and organ damage in burned rats**

Yong-jun Yang ^a,1^,SenSu^a,1^, Yong Zhang ^b,1^, Dan-Wu ^a^, Chao Wang ^b^, Yan-Wei ^a^, Xi Peng ^a,b,c,*^

^a^ Clinical Medical Research Center, Southwest Hospital, Third Military Medical University (Army Medical University), Chongqing China. ^b^ Institute of Burn Research, State Key Laboratory of Trauma, Burns and Combined Injury, Southwest Hospital, Third Military Medical University (Army Medical University), Chongqing China. ^c^ Shriners Burns Hospital, Massachusetts General Hospital, Harvard Medical School, Boston, MA 02114.

^1^ These authors contributed equally to this work.

*Correspondence: pxlrmm@163.com; pxlrmm@tmmu.edu.cn.


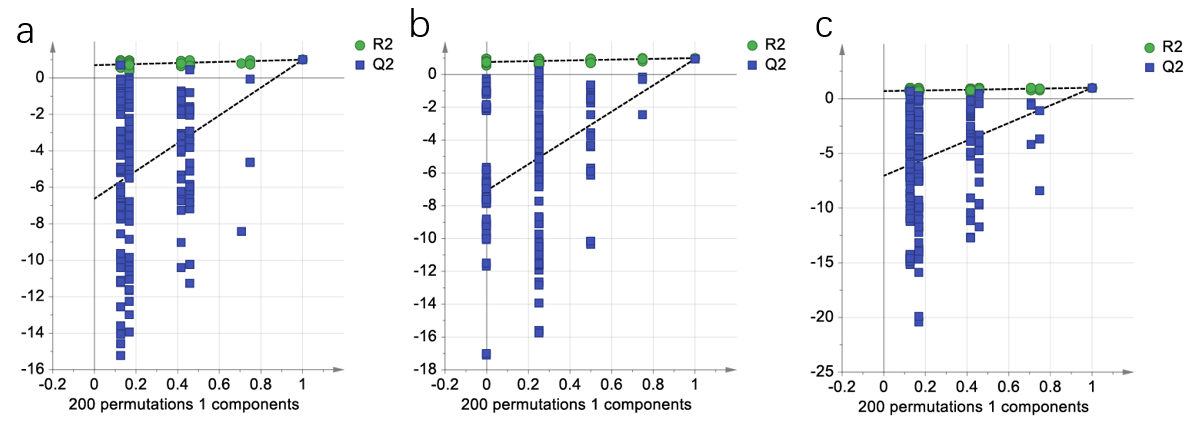


**Figure S1.** OPLS-DA scatter plot from the serum of the statistical validations obtained by 200 times permutation tests：(**a**) 50% and 75%REE-Sup group, (**b**)75% and 100%REE-Sup group, (**c**) 50% and 100% REE-Supgroup


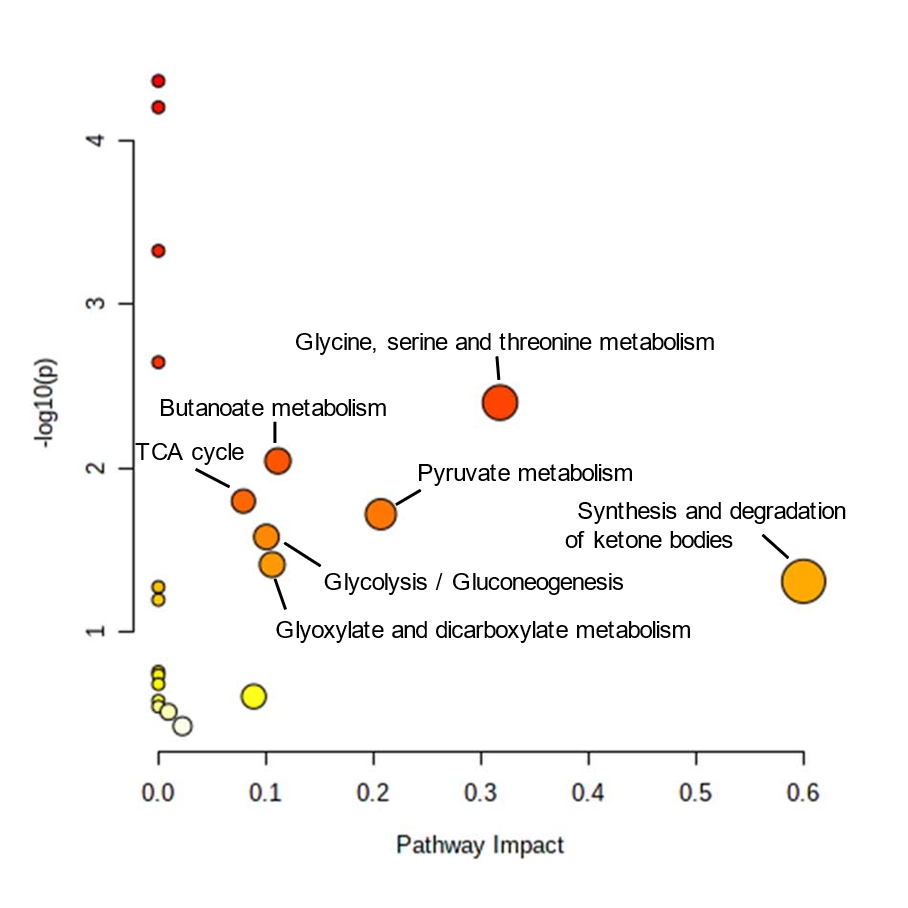


**Figure S2**. Metabolic pathways involved in the functional mechanisms of different energy supplies, including glycine, serine and threonine metabolism, butanoate metabolism, citrate cycle (TCA cycle), pyruvate metabolism, glycolysis/gluconeogenesis, glyoxylate and dicarboxylate metabolism, synthesis and degradation of ketone bodies

**Table S1. Nutritional supplement formula**

| Name | Weight | Energy |
| --- | --- | --- |
| Peptisorb | 100g | 402 Kcal |
| Glucose | 22 g | 88 Kcal |
| Amino acid | 14.35 g | 60 Kcal |
| Medium long chain fatty acid emulsion | 41 mL | 60 Kcal |
| Total | 187.5g | 600 Kcal |

|  |  | | PBD0 | | PBD1 | PBD2 | | PBD3 | | | PBD4 | PBD5 | | PBD6 | PBD7 | | PBD8 | | PBD9 | | PBD10 | | PBD11 | | PBD12 | | PBD13 | | PBD14 | | |
| --- | --- | --- | --- | --- | --- | --- | --- | --- | --- | --- | --- | --- | --- | --- | --- | --- | --- | --- | --- | --- | --- | --- | --- | --- | --- | --- | --- | --- | --- | --- | --- |
| REE  (Kcal/kg/d) | | C | | 262.6±13.9 | 267.9±18.3 | | 258.6±18.4 | | 269.7±12.6 | 254±12.6 | | | 261.9±12.7 | 262.8±15.6 | | 263.4±10.3 | | 249.2±12.6 | | 251.8±15.3 | | 263.8±19.4 | | 268.8±21.6 | | 269.8±24.3 | | 259.3±26.9 | | 267.4±14.3 |  |
|  |  | 100% | | 267.4±10.4 | 281±17.6 | | 264±19.6 | | 238.5±13 | 254±11 | | | 268.3±21 | 286±15 | | 304.2±22.5 | | 307.4±16.3 | | 315.3±18.3 | | 326.6±33.5 | | 329.2±25.1 | | 330.3±18.7 | | 331.3±21.3 | | 333±15.1 |  |
|  |  | 75% | | 255.1±13.2 | 245.4±19.3 | | 259.1±27.1 | | 229.3±16.4 | 263±11 | | | 285.3±24 | 297±17.3 | | 305.6±33.5 | | 302.3±12.2 | | 299.6±17.2 | | 302.3±15.9 | | 305.2±18.3 | | 309.3±17.5 | | 314.4±19.7 | | 318.5±25.7 |  |
|  |  | 50% | | 250.3±19.7 | 247±24.3 | | 231.2±16 | | 220.3±19.3 | 235.5±12 | | | 281.7±16.3 | 315.3±24.3 | | 339.8±22.6 | | 338.1±22.5 | | 340.3±21 | | 339.9±21.1 | | 340.3±18.3 | | 342.4±18.2 | | 344.3±25.3 | | 346.4±21.1 |  |

**Table S2. Assay values of REE in each group of rats from 1-14 days after burn injury**

Note: *C* control group, *100%* 100% REE-Sup group, *75%* 75% REE-Sup group, *50%* 50% REE-Sup group, *REE* resting energy expenditure

**Table S3. The actual amount of energy supplied to each group of rats from 1-14 days after burn injury**

|  |  | PBD0 | PBD1 | PBD2 | PBD3 | PBD4 | PBD5 | PBD6 | PBD7 | PBD8 | PBD9 | PBD10 | PBD11 | PBD12 | PBD13 | PBD14 |
| --- | --- | --- | --- | --- | --- | --- | --- | --- | --- | --- | --- | --- | --- | --- | --- | --- |
| Energysupply  (Kcal/kg/d) | C | 263.1±13.4 | 267.4±18.0 | 258.1±18.7 | 268.2±11.4 | 255.1±11.2 | 261.7±12.2 | 262.2±14.1 | 263.8±10.7 | 249.9±13.1 | 252.2±14.8 | 264.4±18.7 | 269.1±20.7 | 270.8±23.6 | 250.2±25.9 | 268.1±14.2 |
|  | 100% | - | 93.3±5.4 | 132.1±9.3 | 178.4±9.7 | 190.6±7.3 | 201.6±16.2 | 214.2±11.5 | 228.2±16.9 | 308.1±16.2 | 315.8±17.8 | 325.9±32.7 | 328.7±24.9 | 331.1±17.9 | 332.4±20.8 | 332.5±15.1 |
|  | 75% | - | 81.5±6.3 | 129.6±13.9 | 172.2±12.1 | 196.5±7.1 | 214.4±17.2 | 223.1±13.4 | 229.3±25.4 | 226.7±9.1 | 224.7±13 | 226.7±11.9 | 228.9±13.7 | 232±13.1 | 235.8±14.8 | 238.9±19.3 |
|  | 50% | - | 82.5±8.6 | 115.2±7.4 | 110.2±9.8 | 117.7±5.4 | 140.7±8.7 | 157.7±12.6 | 169.9±11.5 | 168.9±10.3 | 169.1±7.8 | 170.4±10.9 | 170.5±9.7 | 171.7±9.2 | 172.5±12.7 | 173.8±10.7 |

Note: The energy supply regimen: Control group (C), given in full amount as measured by REE.100% REE-Sup group (100%), 33% REE on day 1, 50% REE on day 2, 75% REE on days 3-7, 100% REE on days 8-14; 75% REE-Sup group (75%), 33% REE on day 1, 50% REE on day 2, 75% REE on days 3-14;50% REE-Sup group (50%), 33% REE on day 1 REE, days 2-14 50% REE.

**Table S4. Food intake of rats in each group from 1-14 days after burn injury**

|  |  | PBD0 | PBD1 | PBD2 | PBD3 | PBD4 | PBD5 | PBD6 | PBD7 | PBD8 | PBD9 | PBD10 | PBD11 | PBD12 | PBD13 | PBD14 |
| --- | --- | --- | --- | --- | --- | --- | --- | --- | --- | --- | --- | --- | --- | --- | --- | --- |
| Food  （g/kg） | C | 82.1±4.3 | 83.7±5.7 | 80.8±5.8 | 84.3±4 | 79.6±4 | 81.9±3.9 | 82.1±4.9 | 82.3±3.2 | 77.9±3.9 | 78.7±4.8 | 82.4±6 | 84±6.8 | 84.3±7.6 | 77.9±8.4 | 83.6±4.5 |
|  | 100% |  | 29.2±1.8 | 41.3±3.1 | 55.7±2.9 | 59.7±2.5 | 62.9±5 | 66.9±3.4 | 71.3±5.3 | 96.1±5.1 | 98.5±5.7 | 102.1±10.5 | 102.9±7.9 | 103.2±5.9 | 103.5±6.6 | 104.1±4.7 |
|  | 75% |  | 25.6±2 | 40.5±4.3 | 53.7±3.9 | 61.5±2.5 | 66.9±5.5 | 69.7±4.1 | 71.6±7.9 | 70.9±2.8 | 70.2±4 | 70.9±3.7 | 71.5±4.3 | 72.5±4.1 | 73.7±4.6 | 74.6±6 |
|  | 50% |  | 25.8±2.5 | 36.1±2.4 | 34.4±3 | 36.8±1.8 | 44±2.6 | 49.3±3.8 | 53.1±3.5 | 53.1±3.5 | 52.8±2.5 | 53.1±3.3 | 53.2±2.9 | 53.5±2.8 | 53.8±3.9 | 54.1±3.3 |

Note: 1. Food supply weight values for 1-14 days post-burn were discounted according to the energy supply quantity and food weight parameters obtained in Table S3 and Table S1.

2. *C* control group, *100%* 100% REE-Sup group, *75%* 75% REE-Sup group, *50%* 50% REE-Sup group

**Table S5. Amount of protein supplied from 1-14 days after burn injury**

|  |  | PBD0 | PBD1 | PBD2 | PBD3 | PBD4 | PBD5 | PBD6 | PBD7 | PBD8 | PBD9 | PBD10 | PBD11 | PBD12 | PBD13 | PBD14 |
| --- | --- | --- | --- | --- | --- | --- | --- | --- | --- | --- | --- | --- | --- | --- | --- | --- |
| Protein（g/kg） | C | 12.6 ±0.7 | 12.8±0.9 | 12.4±0.9 | 12.9±0.6 | 12.2±0.6 | 13.1±0.6 | 13.1±0.8 | 12.9±0.5 | 11.9±0.6 | 12.1±0.8 | 12.6±1 | 12.9±1.1 | 12.9±1.2 | 11.9 ±1.3 | 12.8±0.7 |
|  | 100% | - | 4.5±0.3 | 6.3±0.5 | 8.6±0.5 | 9.1±0.4 | 9.6±0.8 | 10.3±0.6 | 10.9±0.8 | 14.7±0.8 | 15.1±0.9 | 15.6±1.6 | 15.8±1.2 | 15.8±0.9 | 15.9±1.0 | 15.9±0.7 |
|  | 75% | - | 4.1±0.4 | 5.9±0.7 | 8.2±0.6 | 9.4±0.4 | 10.2±0.9 | 10.7±0.6 | 11.0±1.3 | 10.8±0.5 | 10.8±0.6 | 10.8±0.6 | 11.1±0.7 | 11.1±0.6 | 11.3±0.7 | 11.4±1 |
|  | 50% | - | 4.1±0.4 | 5.8±0.4 | 5.3±0.5 | 5.6±0.3 | 6.7±0.4 | 7.5±0.6 | 8.1±0.6 | 8.1±0.4 | 8.2±0.5 | 8.1±0.5 | 8.1±0.5 | 8.2±0.5 | 8.2±0.6 | 8.3±0.5 |

Note: 1. Calculated and obtained protein supply weights for rats for 1-14 days based on the food weight and protein content parameters obtained in Table S3 and Table S1; Calories per gram of protein are calculated as 4.18 Kcal.

2. *C* control group, *100%* 100% REE-Sup group, *75%* 75% REE-Sup group, *50%* 50% REE-Sup group

**Table S6. Identified metabolites from different groups with S-plot, VIP and *P* value**

| Metabolites | ^1^H Shift(δ) | VIP | *P*-values | Comparison |
| --- | --- | --- | --- | --- |
| Leucine | 0.955(d) 0.965(d) 0.975(d) 1.691(m) 1.707(m) 3.685(dd) 3.753(d) | 3.21 | 0.008 | 50%VS100% |
| Isoleucine | 0.943(t) 1.000(d) 1.008(d) 1.284(m) 1.459(m) 1.961(m) | 1.57 | 0.045 | 50%VS100% |
| Valine | 0.988(d) 1.020(d) 1.040(d) 1.052(d) 2.285(m) 3.570(d) 3.617(d) | 1.56 | 0.035 | 50%VS100% |
| Lactic acid | 1.341(d) 4.108(q) | 1.38 | 0.018 | 50%VS100% |
| Alanine | 1.480(d) 1.492(m) 3.783(q) | 1.97 | 0.027 | 50%VS100% |
| Succinic acid | 2.395(s) | 1.17 | 0.018 | 50%VS100% |
| Creatine phosphate | 3.92(s) 3.051(s) 4.066(s) | 2.29 | 0.020 | 50%VS100% |
| Malonic acid | 3.26(s) | 1.15 | 0.016 | 50%VS100% |
| Glycine | 3.558(s) | 1.60 | 0.040 | 50%VS100% |
| N-acetylated glycoproteins (NAG) | 2.04 (s) | 1.21 | 0.035 | 75%VS100% |
| Trimethylamine | 3.21(s) | 2.51 | 0.023 | 50%VS75% |
| Acetoacetate | 2.273(s) 3.441(s) | 3.49 | 0.007 | 75%VS100% |
| Phosphocholine | 3.218(s) 3.585(m) 4.142(m) | 1.85 | 0.011 | 50%VS75% |
| Betaine | 3.271(s) 3.915(s) | 1.78 | 0.018 | 75%VS100% |
| Glyceraldehyde | 3.60(s) | 6.85 | 0.023 | 75%VS100% |
| Pyruvate | 2.318(s) 2.372(s) | 1.82 | 0.010 | 50%VS75% |

Note: Type of ^1^H Shift(δ): *s* single, *d* doublet, *t* triplet, *m* multiple, *q* quartet, *dd* doublet of doublets

**Table S7. The associated metabolic pathways of the differential metabolites in Table S6, as analyzed using MetaboAnalyst 5.0**

| **Pathway name** | **Hits/Total** | ***P-*value** | **–log(p)** | **Impact** |
| --- | --- | --- | --- | --- |
| Glycine, serine and threonine metabolism | 3/34 | 0.00398 | 2.4005 | 0.31772 |
| Butanoate metabolism | 2/15 | 0.00903 | 2.0445 | 0.11111 |
| Citrate cycle (TCA cycle) | 2/20 | 0.01587 | 1.7995 | 0.07907 |
| Pyruvate metabolism | 2/22 | 0.01907 | 1.7196 | 0.20684 |
| Glycolysis / Gluconeogenesis | 2/26 | 0.02622 | 1.5813 | 0.10044 |
| Glyoxylate and dicarboxylate metabolism | 2/32 | 0.03867 | 1.4127 | 0.10582 |
| Synthesis and degradation of ketone bodies | 1/5 | 0.04888 | 1.3108 | 0.6 |

Note: Total is the total number of compounds in the pathway; Hits is the actually matched number from the user-uploaded data; Impact is the pathway impact value calculated from pathway topology analysis
